# Lung Cancer: New Directions in Senior Patients Assessment

**DOI:** 10.3390/geriatrics9040101

**Published:** 2024-08-01

**Authors:** Anca Iuliana Pîslaru, Sabinne-Marie Albișteanu, Adina Carmen Ilie, Ramona Ștefaniu, Aurelia Mârza, Ștefan Moscaliuc, Mălina Nicoară, Ana-Maria Turcu, Gabriela Grigoraș, Ioana Dana Alexa

**Affiliations:** 1 Department of Medical Specialties II, Faculty of Medicine, “Grigore T. Popa” University of Medicine and Pharmacy, 700115 Iasi, Romania; anca.morosanu2@umfiasi.ro (A.I.P.); adina.ilie@umfiasi.ro (A.C.I.); ramona.stefaniu@umfiasi.ro (R.Ș.); aurelia-m-marza@d.umfiasi.ro (A.M.); ana-maria_turcu@umfiasi.ro (A.-M.T.); grigoras_gabriela@d.umfiasi.ro (G.G.); ioana.alexa@umfiasi.ro (I.D.A.); 2 Department of Oncology, Faculty of Medicine, “Grigore T. Popa” University of Medicine and Pharmacy, 700115 Iasi, Romania; stefan.moscaliuc@yahoo.com (Ș.M.); airmalina@gmail.com (M.N.)

**Keywords:** senior patients, geriatric assessment, frailty, biomarkers, lung cancer

## Abstract

Age is but one significant prognostic factor in lung cancer, influencing survival, treatment response, and outcomes. This narrative review synthesizes findings from searches of 11 leading databases of research studies, systematic reviews, book chapters, and clinical trial reports on lung cancer in senior patients, with a focus on geriatric assessment as well as biomarkers. Key prognostic factors for lung cancer in seniors include biological age, functional capability, physical and psychological comorbidities, frailty, nutrition, status, and biomarkers like DNA methylation age. We identified the most valuable assessments that balance efficacy with quality of life. Optimizing care and improving outcomes with senior lung cancer patients benefits from a tailored therapeutic approach incorporating a complex geriatric assessment. A multidisciplinary collaboration between geriatricians, oncologists, and pulmonologists is crucial.

## 1. Introduction

Lung cancer is a significant concern for senior patients, with half of all cases occurring in this age group [1,2]. Lung cancer symptoms in seniors can be vague and misleading, making it crucial to consider selective screening for this population [1,2]. Despite the potential for aggressive therapy to enhance survival, the percentage of senior patients undergoing treatment tends to decline [3]. Age alone should not prevent older lung cancer patients from receiving optimal evaluation, treatment, and care [4].

We intended to bridge knowledge gaps and highlight areas where more research is needed, particularly in understanding how age-related changes in pharmacokinetics and pharmacodynamics affect treatment efficacy and toxicity. By consolidating data on prognostic and predictive factors specific to senior lung cancer patients, this review can guide clinicians in personalizing treatment plans, balancing the goals of prolonging survival and preserving quality of life.

Lung cancer is the leading cause of cancer-related mortality worldwide, causing more than 2.2 million new cases and more than 1.8 million deaths in 2020, according to the World Health Organization. Lung cancer rates are very low below the age of 40, rising progressively until the age of 80, after which they decrease [5]. The leading risk factor for lung cancer remains active tobacco smoking, being responsible for 80–95% of cases in males and 50–90% in females. Passive smoking is also an important risk factor, comprising 20–50% of lung cancer cases in non-smokers. Other risk factors include occupational carcinogens such as asbestos, radon, and diesel fumes [6].

Aging is a significant factor in the development and treatment of lung cancer. This is due to physiological changes in the aging lung, which increase susceptibility to lung diseases [7]. However, age itself is not a significant prognostic factor for overall survival and response to treatment [8]. Senior patients, particularly those over 80, are less likely to receive surgery or radiation and have lower survival rates, with older patients less likely to receive active treatment [9,10].

The objective of this review is to evaluate specific biomarkers in the context of their prognostic and predictive value for lung cancer treatment in the senior population.

Our aim is also to delineate the unique aspects of cancer prognosis and treatment efficacy within this age group, recognizing that seniors often have different comorbidities, physiological reserves, and treatment tolerance than younger cohorts. Additionally, such a review underscores the importance of geriatric assessment in treatment planning and the need for a multidisciplinary approach in managing lung cancer in seniors. 

This review hopes to break new ground by demonstrating that prioritizing key prognostic factors over chronological age can help clinicians improve prediction and prioritize treatment alternatives.

Many senior patients are frail due to geriatric syndromes and age-related conditions. Lung neoplasm requires a complex approach and special attention due to its devastating, accelerating clinical impact. Senior patients often present with more advanced disease, experience different side effects than the general population, and may exhibit decreased organ function and a higher incidence of comorbidity. Multiple factors outweigh chronological age in predicting outcomes and prioritizing treatment alternatives. Certain seniors, as well as younger patients, respond to chemotherapy and other treatments, and yet senior patients with lung cancer often face undertreatment, despite the demonstrated benefits of appropriate care. Older patients should not be automatically excluded from treatment based solely on their age.

### Literature Search and Selection Methodology

We searched 11 leading databases for peer reviewed research studies, systematic reviews, book chapters, and clinical trial reports on lung cancer on senior patients, with a focus on geriatric assessment as well as the safety and efficacy of biomarkers. For the pharmaceutical and biomedical literature, we searched PubMed/MEDLINE, EMBASE, Web of Science, Scopus, Cancer.gov, ClinicalTrials.gov, and Science Direct. In addition, for biomarkers in senior patients with lung cancer, we searched Cochrane Library and BioMed Central. We utilized Google Scholar for preliminary searches and open access articles.

We used specific keywords and phrases like “lung cancer biomarkers in seniors”, “lung cancer elderly”, “geriatric oncology lung cancer”, “biomarker identification in lung cancer aging, “screening tools for geriatric outcomes in lung cancer”, “assessments for geriatric outcomes after lung cancer therapy”, “geriatric lung cancer characteristics”, and “geriatric evaluation in lung cancer” to refine the search results. 

We focused on the most recent and high-impact lung cancer research. We narrowed our search down to the relative benefit of assessment versus traditional factors like chronological age and prognostic and predictive biomarkers in senior patient management. 

## 2. Biomarkers and Biological Reactivity in Lung Cancer Senior Patients

### 2.1. Biomarkers Used in the Diagnosis, Prognosis, and Treatment of Lung Cancer

In clinical trials, biomarkers provide essential endpoints that can help assess the efficacy of new drugs and treatment strategies. They also play a crucial role in reducing the time and cost associated with drug development by facilitating patient selection for clinical trials, ensuring that only those most likely to benefit are enrolled.

Understanding how these biomarkers might vary in senior patients, and their implications for prognosis and treatment, is a rich development with much unexplored territory.

Key biomarkers utilized in clinical practice and research for diagnosis, prognosis, and treatment guidance in lung cancer in the general population are shown in the following table (Table 1):

### 2.2. Biomarkers in Lung Cancer Specific to Geriatric Patients

In the senior population with lung cancer, various biomarkers, including age, functional status, and disease stage, have been identified. The DNA methylation age, a biomarker of biological aging, is associated with increased lung cancer risk, particularly in older individuals [11,12]. The macrophage migration inhibitory factor (MIF) may play a role in age-related lung diseases, including lung cancer [13]. For senior lung cancer patients, biomarker testing has as much potential to improve the targeting of treatment and subsequent survival as for their younger counterparts. 

#### 2.2.1. MicroRNA

MicroRNA molecules are very useful in the early diagnosis and monitoring of senior patients with lung cancer. They can support prognosis and help predict treatment response. Despite obstacles with cost and detection, they are easy to sample through liquid biopsy.

MicroRNAs (miRNA) are a class of small, non-coding RNA sequences, measuring 18 to 25 nucleotides in length, with a role in the regulation of gene expression. They are negative regulators, being able to degrade or block the translation of mRNAs. As such, they can regulate multiple cellular processes such as cell growth and apoptosis, and even tumor progression. miRNAs are excreted into the bloodstream, being transcribed after changes in the regulation of neoplastic cells, which means that they can be obtained from liquid biopsies. Liquid biopsies are simple to perform and are easily repeatable; however, miRNA detection can be challenging. Methods generally used for detecting miRNAs are RT-PCR or Northern blotting, each of them having different limitations, such as a low detection efficiency for Northern blot or a high cost for RT-PCR [14]. 

#### 2.2.2. Circulating Tumor DNA

Similarly to miRNA, another biomarker easily sampled through liquid biopsy is circulating tumor DNA (ctDNA). The blood contains numerous fragments of non-encapsulated extracellular DNA, also known as cell-free DNA, being released by apoptotic and necrotic cells. In patients with cancer, those fragments can come from neoplastic cells, thus naming them ctDNA [2]. Being prevalent in most patients with advanced solid tumors, ctDNA has been one of the most studied biomarkers in recent years, having many applications in the clinical management of neoplasms, even more so in NSCLC management. The preferred method of ctDNA detection is through Next-Generation Sequencing (NGS), which has become a mandatory component in lung cancer management, being a standard in the detection of ALK, EGFR, and BRAF mutations amongst many others, which helps in making treatment decisions [7]. The main difficulty in detecting ctDNA in serum or plasma is that it has a very short half-life, between fifteen minutes and two and a half hours [15].

Being able to easily identify small fragments of tumor DNA in the bloodstream of patients with cancer presents many opportunities, such as treatment guidance, monitoring treatment response, and identifying resistance mutations. 

ctDNA detection is therefore useful in finding lung cancer patients that are likely to relapse after initial curative treatment and who may benefit from further treatments. While present studies have not proven that the absence of ctDNA in post-treatment lung cancer patients exempts them from the need for adjuvant treatment, further clinical trials may be able to determine a balance of benefit/harm. Senior patients are at a higher risk of toxicity and mortality with cancer treatments, often due to numerous comorbidities and dysfunction in aging organs. As such, maximizing therapy benefits and lowering treatment risks, the base principle in treating any cancer patient, are even bigger challenges in senior patients [16].

#### 2.2.3. DNA Methylation Age of Blood

DNA methylation at CpG sites offers a promising biomarker for assessing the risk of aging-related lung cancer. The accumulation of somatic mutations within cells with chronic cell cycling that leads to genomic instability is considered a hallmark of both cancer and aging. Epigenetic clocks are considered to be reliable markers of biological aging based on DNA levels. They have the ability to predict age-related diseases, like cancer, and outcomes, including frailty and mortality [17].

An accurate aging biomarker based on levels of DNA methylation was recently developed. Also called the “epigenetic clock”, it assesses the biological age of human cells, tissues, and organs. It is potentially useful for assessing the risk of aging-related diseases such as lung cancer. Beyond smoking, chronological age stands as a significant risk indicator for lung cancer. Lung cancer’s link with aging is acknowledged, displaying a sharp increase in incidence across the lifespan that peaks in the seventh to eighth decades. The association between lung cancer and age is partially attributed to the progressive accumulation of unrepaired damage from exposure to carcinogens, such as those in cigarette smoke, combined with the decline in immune function and increased age-associated cellular senescence. However, the rate of these age-related changes varies significantly among individuals, suggesting that chronological age may not accurately reflect the biological aging processes influencing lung cancer susceptibility. Considering smoking’s role as an aging accelerator and lung cancer’s link with aging, an epigenetic measure of age acceleration could shed light on individuals most at risk of developing lung cancer [12].

## 3. Performance Status, Nutritional Status, Albumin Levels, and Individualization of Therapeutic Approach

Laboratory markers such as albumin offer an affordable and accessible way to assess the nutritional status of individuals with cancer. Moreover, albumin acts as a negative acute-phase reactant, with levels typically decreasing in chronic inflammatory conditions like cancer. 

Although initial exposure to harmful agents such as carcinogens might boost albumin production, in advanced cancer stages, we often see a marked reduction in albumin levels. This is due to malnutrition and the suppressive effects of cytokines and chemokines, including CRP and IL-6, on liver function. Consequently, albumin levels emerge as a biologically plausible biomarker that can simultaneously indicate the nutritional and inflammatory status of cancer patients, in whom lower albumin levels may signal a more progressed cancer stage and a poorer prognosis. This evidence underscores the significance of albumin levels as a prognostic biomarker in senior cancer patients, surpassing other potential biomarkers related to liver function [18].

ECOG performance status (PS) and albumin levels at diagnosis emerged as the most significant predictors of OS for patients in the BSC category. The findings indicated that patients with favorable PS and high albumin levels experienced longer survival periods, even when opting for BSC as their lung cancer treatment approach. Patients aged 81 years and older appeared to have a longer OS in the chemotherapy group compared to the BSC group. With increasing age, the diversity and individual differences among older people become more pronounced, underlining the need for attentive and tailored treatment planning for those aged 81 and above [19].

Hypoalbuminemia and nutritional status were significantly linked to the premature cessation of chemotherapy, and patients without hypoalbuminemia experienced a notable survival advantage from chemotherapy. Independently, hypoalbuminemia was associated with poorer survival outcomes within the chemotherapy group, indicating that patients with hypoalbuminemia had a poorer prognosis, regardless of whether they underwent chemotherapy. Previous epidemiological research examining the relationship between serum albumin levels before treatment and survival in non-small-cell lung cancer (NSCLC) found that higher serum albumin levels were correlated with improved survival [20].

Thus, the anticipated rise in lung cancer cases among older individuals underscores the importance of obtaining thorough information and evaluating both the patient’s overall health status and their treatment preferences comprehensively [19].

## 4. Geriatric Lung Cancer Screening Tools

### 4.1. Clinical Toolkits and Assessment Tools—Geriatric 8, Identification of Seniors at Risk—Hospitalized Patients (ISAR-HPs), and Comprehensive Geriatric Assessment (CGA)

Clinical toolkits and assessment tools commonly used to measure geriatric rehabilitation outcomes, particularly in the context of lung cancer, are discussed below. These tools can provide comprehensive insights into the functional status, quality of life, and rehabilitation progress of senior patients, particularly those undergoing treatment for lung cancer [21].

### 4.2. The Role of Geriatric Screening Instruments in Lung Cancer Assessment

The Geriatric 8 (G8) screening tool is an assessment method designed to evaluate the health status of older patients to identify those at risk of poor outcomes [22]. It encompasses various domains, including food intake, weight loss, mobility, neuropsychological problems, body mass index, medications, self-perceived health status, and age. The G8 tool is particularly useful in the oncology setting, helping healthcare professionals to determine which senior cancer patients may benefit from a more comprehensive geriatric assessment [23]. By identifying vulnerabilities early, the G8 screening tool aids in tailoring cancer treatment plans to individual needs, enhancing care quality and potentially improving survival and quality of life for older patients [24] (Table 2).

The comprehensive geriatric assessment (CGA) is a method frequently employed to evaluate the medical, psychological, and functional capacities of older individuals. 

Two proposed tools for this purpose include the Geriatric 8 (G8) and the Identification of Seniors at Risk—Hospital Patients (ISAR-HPs). A notable aspect of these instruments is their emphasis on nutritional status, which accounts for nearly half (46%) of the total score. They assess other key areas such as mobility, neuropsychological issues, medication usage, self-perceived health, and age. The G8’s highest possible score is 17 points, with a score below 14 considered impaired. The ISAR-HPs is an ascending scale, where the maximum score is four, and scores of two or higher indicate impairment [25].

The Groningen Frailty Indicator (GFI) has been evaluated as a screening tool for frailty in older cancer patients, including those with lung cancer. 

The G8 score has been identified as an independent prognostic indicator for overall survival (OS), similar to the ECOG performance status (PS). These results imply that the G8 questionnaire could serve as a practical instrument in guiding treatment decisions by predicting prognosis and helping to avoid the administration of unsuitable anti-cancer treatments towards the end of life, taking into consideration quality of life [26].

When feasible, a full CGA evaluating medical, cognitive, functional, and psychosocial status along with interrelated complexities can aid targeting and reveal treatment progress at a level of detail well beyond that of the Geriatric 8 and ISAR-HPs, laying the foundation for truly personalized medicine. 

### 4.3. Other Screening Tools and Assessments

Functional Assessment of Cancer Therapy—Lung (FACT-L) is a specialized questionnaire designed to assess quality of life in patients undergoing treatment for lung cancer, addressing physical, social, emotional, and functional well-being. The FACT-L/LCS questionnaire measures physical, social, emotional, and functional well-being, as well as lung cancer-related symptoms and regret of smoking [27]. A brief symptom index, the Functional Assessment of Cancer Therapy—Lung Symptom Index-12 (FLSI-12), has also been validated for use in advanced-stage lung cancer patients, showing good reliability and validity [28].

Karnofsky Performance Status (KPS) is a predictor of survival in senior patients with limited-disease small-cell lung cancer [29]. It is also a key factor in overall survival of senior patients with muscle-invasive bladder cancer, with a KPS of 90 or greater providing a significant survival advantage [30]. 

Pulmonary function tests (PFTs): Essential for assessing lung function in patients with lung cancer, PFTs provide vital information about respiratory status and capacity, informing rehabilitation strategies [31]. Senior patients can achieve spirometry and diffusion-capacity quality scores comparable to younger adults. This indicates the reliability of pulmonary function tests in this population [32], particularly if age-related changes in pulmonary function, such as a normal decline in values, are considered when interpreting these tests [33]. The preoperative evaluation of pulmonary function is crucial for senior lung cancer patients, and postoperative physical therapy may be necessary [34,35]. Pulmonary rehabilitation is a valuable intervention for senior patients with pulmonary diseases, including lung cancer [35].

The 6-Minute Walk Test (6MWT) is a practical test used to evaluate a patient’s endurance and functional capacity. It is particularly relevant for assessing the impact of lung cancer and its treatment on physical stamina [36]. 

The Patient-Reported Outcomes Measurement Information System (PROMIS) is an adaptable system of patient-reported outcome tools that assesses several domains of health and well-being. The PROMIS allows for tailored assessments in geriatric rehabilitation contexts, including those with lung cancer [37]. 

The Triage Risk Screening Tool (TRST) is a quick, efficient method used primarily in healthcare settings to identify patients at risk of functional decline or adverse outcomes. It is designed to be used upon a patient’s admission to facilitate early intervention and appropriate resource allocation [38]. The tool assesses various risk factors, including cognitive status, mobility, and prior healthcare utilization, to categorize patients based on their need for further evaluation, particularly in identifying senior patients in need of social service intervention [39]. 

## 5. Discussion

### 5.1. The Implications of Biomarkers in Lung Cancer Management

Biomarkers play a crucial role in the paradigm shift towards personalized medicine, especially in the context of lung cancer, where they enable more precise diagnosis, treatment, and monitoring [40]. They can be DNA, RNA, proteins, peptides, or metabolites, reflecting the state of disease or the effects of treatment [40,41].

Predictive biomarkers are crucial for determining the likelihood of a patient’s response to a specific therapy. They are instrumental in personalized medicine, helping clinicians choose the most effective treatment based on the biomarker’s presence or absence in the tumor. The use of predictive biomarkers in lung cancer is a rapidly evolving field, with potential for a significant impact on treatment decisions. Key biomarkers include EGFR mutations, ALK and ROS1 rearrangements, and PD-L1 expression, which can help guide targeted and immunotherapy choices [17,42]. Tissue and circulating biomarkers are also gaining importance in predicting treatment response and prognosis [43]. The role of PD-L1 expression and tumor mutational load in selecting patients for immune checkpoint inhibitors is particularly promising [44]. However, the overall impact of these biomarkers on survival is still being explored, particularly with respect to senior patients. 

Prognostic biomarkers provide general outcome information. Predictive biomarkers of response to treatment guide therapy decisions and enhance the precision of lung cancer treatment. Understanding these differences is essential for interpreting clinical trial data and applying it to patient care, ultimately improving outcomes in lung cancer management. The identification of prognostic biomarkers in lung cancer is crucial for guiding treatment decisions and improving patient outcomes. For senior patients, the potential of driver biomarkers, such as EGFR and ALK, is promising. Driver biomarkers predict the response to targeted therapies. Prognostic markers such as proteases and genetic abnormalities, particularly cathepsin B and altered p53 expression, show similar promise for senior lung cancer patients [11,17,45,46].

Senior patients with lung cancer present with different symptoms and comorbidities than younger patients, and they may benefit from tailored treatment strategies [12,47,48,49,50,51,52,53]. DNA methylation age, a biomarker of biological aging, is particularly associated with increased risk of lung cancer in older individuals [12]. While age, performance status, and disease stage are commonly used to guide therapeutic decisions in lung cancer, more sensitive biomarkers are needed [48,54,55,56,57,58,59,60,61,62,63].

#### 5.1.1. Biomarkers Used in the Diagnosis, Prognosis, and Treatment of Lung Cancer 

The BATTLE-1 trial, a landmark study in lung cancer, which included senior patients, demonstrated the feasibility of a personalized approach to clinical trials, using real-time biomarker analysis to guide treatment selection [64,65].

The BATTLE-1 trial was the inaugural prospective, adaptively randomized trial in heavily pretreated NSCLC patients that required tumor profiling by “real-time” biopsies. This study marked a significant advancement in the journey toward tailored lung cancer therapy by incorporating real-time molecular laboratory results to identify distinct patient cohorts for personalized treatment. BATTLE-2 was launched in 2015, and its literature added -1 to the name of the inaugural trial [66].

Recent analyses on biomarkers in non-small-cell lung cancer (NSCLC) following the BATTLE-1 trial have explored various predictive and prognostic biomarkers to enhance personalized therapy. One study analyzed clinical trial and real-world data to evaluate the clinical utility of PD-L1 and tumor mutational burden (TMB) as predictive biomarkers for PD-1 and PD-L1 checkpoint inhibitor response. It found that high PD-L1 expression and high TMB were significantly associated with a durable response to checkpoint inhibitors (So et al., 2023) [67]. Another study assessed baseline immune-suppressive biomarkers like neutrophil-to-lymphocyte ratio (NLR), platelet-to-lymphocyte ratio (PLR), and lymphocyte-to-monocyte ratio (LMR) in predicting outcomes for NSCLC patients treated with plinabulin and docetaxel. It suggested that these biomarkers could serve as prognostic markers to identify patients likely to benefit from the combination (Feinstein et al., 2023) [68]. Moreover, comprehensive biomarker analyses of sorafenib in the BATTLE-1 trial revealed significant correlations between clinical benefits and specific genetic profiles, identifying subgroups that may derive a clinical benefit from sorafenib [69].

Recent studies have investigated the significance of biomarkers in geriatric patients with non-small-cell lung cancer (NSCLC). These analyses focus on how biomarkers can predict therapy responses and survival outcomes in senior populations, which is crucial given the different physiological profiles and treatment tolerances in geriatric patients. One study examined immunosenescence biomarkers, highlighting that senior NSCLC patients with higher frequencies of specific T cell populations had better responses to the CIMAvax-EGF cancer vaccine [38]. Another study evaluated the prognostic value of biomarkers like HE4, CEA, SCCA, and CY21-1 in a Chinese senior population, finding that these markers were significantly associated with increased NSCLC risk and could help in early detection (Guo et al., 2021) [70]. Additionally, eosinophil count and immune-related adverse events (irAEs) have been identified as predictive biomarkers of survival in senior NSCLC patients treated with immune checkpoint inhibitors (Giommoni et al., 2023) [71].

However, the identification of predictive biomarkers for EGFR therapy, a key target in lung cancer treatment, remains a challenge due to conflicting data [72]. In the context of lung cancer chemoprevention, miR-34c has shown promise as a potential biomarker, correlating with histology and treatment response [73]. These studies collectively highlight the potential of biomarkers in guiding personalized treatment and chemoprevention strategies for lung cancer in geriatric patients.

In lung cancer, biomarkers have revolutionized patient care in the general population, offering insights that guide therapeutic decisions and prognoses. For instance, mutations in the EGFR gene, alterations in the ALK gene, and the presence of PDL1 protein can dictate the choice of targeted therapies, enhancing treatment efficacy and minimizing toxicity [74]. By identifying specific genetic mutations or protein expressions in lung cancer cells, clinicians can prescribe treatments that are more likely to be effective for the individual patient, such as tyrosine kinase inhibitors for patients with EGFR mutations [75].

The molecular characterization of lung cancer has led to the identification of novel biomarkers, such as epidermal growth factor receptor mutations and anaplastic lymphoma kinase translocations, which are essential for targeted molecular therapies [76]. These biomarkers, including CYFRA 21-1, NSE, ProGRP, SCC, CEA, tumor M2-PK, CRP, LDH, tumor-suppressor genes, and oncogenes, CA125, CgA, NCAM, and TPA, are useful in the diagnosis and clinical management of lung cancer [77]. Furthermore, the identification of sensitive biomarkers, such as those identified through molecular and genetic studies, is critical for risk stratification and therapeutic decision-making in lung cancer [11].

Moreover, biomarkers in lung cancer facilitate risk stratification and can help in screening high-risk populations, potentially leading to earlier diagnosis and improved outcomes [11]. They also aid in monitoring disease progression and detecting recurrence, allowing for timely adjustments in treatment plans [78]. In the era of immunotherapy, biomarkers like tumor mutation burden (TMB) and microsatellite instability (MSI) have gained prominence, helping to identify patients who are more likely to respond to immune checkpoint inhibitors [43].

#### 5.1.2. Biomarkers in Lung Cancer Specific to Geriatric Patients

- MicroRNA

Multiple families of miRNAs have been studied and are currently being used for their diagnostic role or as prognostic biomarkers. For example, it is now known that an overexpression of miRNAs from the hsa-miR-21 family inhibits the expression of various genes, causing a decrease in apoptosis and an increase in cell proliferation. Studies have been able to link the over/under expression of certain miRNAs with a lower overall survival of patients with forms of lung cancer. The elevated expression of miR-146b and miR-155 is associated with poor survival in squamous cell carcinoma, while higher levels of hsa-miR-10b found in plasma are associated with improved survival in patients with NSCLC [15,79].

mRNA biomarkers have gained attention due to their role in gene expression regulation. Specific mRNA profiles can help in the early detection, prognosis, and monitoring of lung cancer. Techniques like RNA sequencing (RNA-Seq) enable the identification of differentially expressed mRNAs that can serve as biomarkers. In geriatric patients, mRNA biomarkers can aid in the early detection of lung cancer when traditional diagnostic methods might be less effective. For example, mRNA signatures from non-invasive samples (e.g., blood or sputum) can be used to identify lung cancer at an early stage, which is crucial for senior patients who may have comorbidities that complicate invasive procedures. mRNA expression profiles can provide insights into the tumor’s behavior, helping to predict the aggressiveness of the cancer and the likely response to specific treatments. This information can be particularly useful in geriatric patients, allowing clinicians to tailor treatments that minimize side effects while maximizing efficacy. Monitoring mRNA levels can help track disease progression and detect recurrences early. This ongoing monitoring is crucial in geriatric patients, where the timely adjustment of treatment plans can significantly impact outcomes [80].

- Circulating Tumor DNA

In a study by Chaudhuri et al. involving patients with stages I to III lung cancer who had undergone radiotherapy or chemo-radiotherapy, it was found that 94% of patients that presented ctDNA post-treatment in their blood had subsequent recurrences [10]. In another study by D. Gale et al., plasma samples for ctDNA sequencing were collected before, during, and after treatment in patients with non-small-cell lung cancer (NSCLC). Patients that had ctDNA detected in a blood sample collected in a timeframe of 2 weeks to 4 months from the end of treatment had recurrence of their primary tumor and shorter overall survival [81].

- DNA Methylation Age of Blood

The concept of “age acceleration”, defined as the discrepancy between predicted and chronological age, has been recognized in recent epidemiological and clinical research as an indicator of biological aging. Research has explored the link between age acceleration and mortality, and a few studies have demonstrated an association with cancer risk. In one study involving 43 participants, a 50% increase in lung cancer risk was noted for every five-year increment in age acceleration. Another study, with 132 cancer cases, found that age acceleration correlated with a higher risk of cancer and reduced survival time post-diagnosis [16].

A study involving 2029 women from the Women’s Health Initiative investigated if initial “intrinsic epigenetic age acceleration” (IEAA) levels could predict future lung cancer occurrence. During the almost twenty-year follow-up, 43 cases of lung cancer were identified. The findings indicate that standardized IEAA measures were significantly linked to the incidence of lung cancer. Additionally, a possibly stronger link was highlighted in individuals aged 70 or above, or among current smokers. These results imply that IEAA could serve as an effective biomarker of biological aging that can help determine susceptibility to lung cancer among senior patients in particular [12].

The Women’s Health Initiative study’s findings reveal that an aging acceleration rate one standard deviation above the average (standardized IEAA = 1) correlates with up to a 2.5 times higher risk of lung cancer onset, meaning that individuals with an epigenetically older age profile have a higher likelihood of lung cancer occurrence. Conversely, according to the predictive model from this study, only 5% of senior smokers displaying a negative age acceleration (with a standardized IEAA of −1) are expected to develop lung cancer within the next decade. In senior smokers with an average age acceleration (IEAA of 0), 12% are predicted to develop lung cancer, escalating to 25% for those with a positive age acceleration (standardized IEAA of 1). Furthermore, the predictive strength of IEAA for lung cancer incidence is most pronounced in those aged 70 and above. Considering the high mortality associated with lung cancer, the early identification of at-risk individuals is crucial [12].

The epigenetic clock refers to changes in DNA methylation patterns that correlate with biological aging. These changes can also reflect the impact of cancer and its progression. Epigenetic modifications can influence gene expression without altering the DNA sequence, and their patterns can serve as potential biomarkers for lung cancer. Epigenetic biomarkers can help distinguish between age-related changes and cancer-specific alterations. This distinction is vital in seniors, where age-related changes might mask early cancer signs. Measuring the biological age of tissues through the epigenetic clock can help predict how a patient might tolerate and respond to treatments. For instance, a patient with a “younger” epigenetic age might better withstand aggressive treatments compared to someone with an “older” biological age, despite being the same chronological age. Changes in epigenetic markers over time can also be monitored to assess how the cancer and the patient are responding to treatment, providing a dynamic view of the disease state.

Incorporating mRNA and epigenetic biomarkers into clinical practice offers promising advancements in the personalized treatment of lung cancer, especially for geriatric patients. These biomarkers can enhance early detection, inform treatment decisions, and provide ongoing monitoring, all of which are critical for managing lung cancer in seniors with multiple health challenges [82,83].

### 5.2. Performance Status, Nutritional Status, Albumin Levels, and Individualization of Therapeutic Approach

Nutritional status plays a critical role in determining the overall condition of patients and is intimately linked to their survival following cancer treatments, as evidenced by recent studies [18,19].

As mentioned before, certain clinical studies exclude the participation of older individuals in poor physical condition. Research has indicated an increase in adverse events during standard treatment in senior patients. Given the variability in health status among older patients, treatment should be tailored based on their level of fitness, frailty, or susceptibility rather than chronological age [19].

Numerous investigations involving patients undergoing chemotherapy, surgery, targeted therapy, or radiotherapy have shown that lower albumin levels in cancer patients are associated with reduced overall survival (OS) and progression-free survival (PFS) [18].

A study made by Seto in 2021 enrolled a total of 124 lung cancer patients with confirmed wild-type EGFR, with an age between 75 and 94 years, who voluntarily chose between chemotherapy and palliative care. The retrospective analysis considered factors including age, sex, ECOG performance status (PS), TNM stages, and overall survival (OS). Patients undergoing chemotherapy were in an advanced cancer stage, whereas those opting for palliative care could be at any cancer stage [19].

Among the 124 patients with non-small-cell lung cancer (NSCLC), 75 were provided with best supportive care (BSC) without undergoing any targeted treatment for lung cancer. The remaining 49 patients were treated with either single-agent chemotherapy or combination chemotherapy with platinum-based drugs, including the use of immune checkpoint inhibitors (ICIs) [19].

The study explored the link between various clinical factors and overall survival (OS). In analyzing how age impacts median OS among patients who opted for best supportive care (BSC), there was no significant difference between those 80 years old and younger and those older than 81 years. Median OS was 25 and 15 weeks, respectively. This indicates that age at diagnosis does not significantly affect survival in patients receiving BSC. Furthermore, patients with an ECOG performance status (PS) of 0 or 1 at diagnosis experienced a longer OS compared to those with a PS of 2 or higher, with median OS of 60 weeks versus 15 weeks, respectively. Furthermore, patients diagnosed with an albumin level of ≥3.0 g/dL experienced a longer overall survival (OS) compared to those with an albumin level of <3.0 g/dL. Specifically, the median OS was 32 weeks for patients with higher albumin levels, as opposed to 13 weeks for those with lower levels [19]. Thus, we see that chronological age is not a strong predictor of potential for treatment.

It is important to note that older patients should not be automatically excluded from receiving effective treatments based solely on their age. Prior research indicates that senior individuals with favorable PS can undergo chemotherapy as effectively as younger patients and achieve comparable outcomes; therefore, the prognosis with chemotherapy generally surpasses that with BSC alone. When determining the course of treatment post-lung cancer diagnosis—whether to proceed with BSC or to opt for surgery, radiation, or chemotherapy—medical professionals should meticulously assess the adequacy of BSC in light of existing guidelines, keeping in mind that there are no definitive rules for choosing BSC over active interventions [19].

When determining whether to administer active treatment to senior patients, it is crucial to consider if they are more at risk of dying from lung cancer than from age-related conditions. In the previous study, members of the BSC group with lower albumin levels exhibited shorter survival times. Indeed, patients with albumin levels of ≥3.0 and those with a favorable performance status (PS of 0 or 1) might experience extended survival periods, even if they opt for BSC as their lung cancer treatment strategy. Given the expected increase in the number of older lung cancer patients in the future, providing comprehensive information and thoroughly assessing the patient’s overall health condition and preferences are essential [19].

A study made by Ikeda in 2017 highlighted three critical clinical findings. Firstly, in senior patients with poor performance status (PS), the overall survival (OS) was superior in the chemotherapy group compared to the best supportive care (BSC) group. Secondly, the survival advantage of chemotherapy was more significantly influenced by the number of treatment cycles administered rather than the choice between single-agent therapy and carboplatin-doublet therapy. Lastly, low albumin levels (hypoalbuminemia) posed a risk not just for the early discontinuation of chemotherapy but also as an independent prognostic factor within the chemotherapy group [20].

### 5.3. The Value of Clinical Toolkits and Assessment Tools in Managing Senior Patients

Clinical toolkits and assessment tools are indispensable in the management and care of senior patients with lung cancer, addressing unique challenges posed by this demographic. Comprehensive geriatric assessment is a key component, evaluating multiple domains such as functional status, comorbidities, cognition, nutrition, and psychosocial factors to tailor cancer treatment and supportive care.

#### 5.3.1. The Role of Geriatric Screening Instruments in Lung Cancer Assessment

With the global population getting older, there has been a rise in lung cancer cases among seniors. According to the latest information from the United States’ Surveillance, Epidemiology, and End Results program, nearly half (47%) of all lung cancer patients are aged 70 or older. However, the current treatment recommendations come from studies involving healthy older individuals, making it challenging to apply these guidelines in everyday clinical practice where the senior population is often frail and heterogenous [26].

Clinical toolkits and assessment tools are indispensable in the management and care of senior patients with lung cancer, addressing unique challenges posed by this demographic. Comprehensive geriatric assessment is a key component, evaluating multiple domains such as functional status, comorbidities, cognition, nutrition, and psychosocial factors to tailor cancer treatment and supportive care [22].

Comprehensive frailty assessment instruments, such as the G8 questionnaire, evaluate frailty, a critical determinant of treatment approach and prognosis in senior lung cancer patients. Pain assessment tools ensure effective pain management, a fundamental aspect of palliative care. Pulmonary function tests are tailored to assess the impact of lung cancer and potential treatments on respiratory function. Lastly, patient-reported outcome measures (PROMs) allow for the integration of the patient’s voice in evaluating the effectiveness and side effects of treatments, ensuring patient-centered care. These toolkits and tools collectively inform a multidisciplinary approach to optimize outcomes and enhance quality of life for senior lung cancer patients [22,31].

Senior patients with lung cancer often face undertreatment, despite the potential benefits of appropriate care [84]. Geriatric assessment is crucial in determining the most suitable treatment, taking into account the patient’s unique strengths and weaknesses [85]. However, the effectiveness of geriatric assessment in stratifying risk for older adults with lung cancer is debated, with some studies suggesting that it may not be a reliable prognostic tool [86]. The high symptom burden and prevalence of comorbidities in this population further complicate treatment decisions [87]. Despite these considerations, the Geriatric 8 (G8) frailty screening tool is used in medical practice to determine the degree of risk in frail patients. 

The Geriatric 8, a toolkit for measuring geriatric rehabilitation outcomes, was developed and tested for its acceptability and data quality [88,89]. The Geriatric 8 tool, along with the Flemish version of the Triage Risk Screening Tool, was found to be effective in identifying older cancer patients with a geriatric risk profile, and in predicting functional decline and overall survival [90,91].

The Geriatric 8 tool could be a valuable resource in the treatment decision-making process for this patient population. The Geriatric 8 screening tool has been found to be a useful prognostic tool for senior patients with lung cancer, particularly in predicting overall survival and functional decline [25,26,92]. It has also been shown to have good screening properties for identifying patients who could benefit from comprehensive geriatric assessment [22].

At the same time, the identification of risk for hospitalized seniors requires the application of rigorous protocols to which clinicians should also resort in current practice [25].

Certain aspects of the CGA have been found to be helpful in predicting toxicity and functional decline. The International Society of Geriatric Oncology recommends using the CGA for senior cancer patients to identify those who are sufficiently healthy to undergo standard treatments. However, the CGA requires a significant amount of time to complete [26]. On the other hand, the G8 questionnaire offers a swift and straightforward alternative, taking less than 5 min to administer and aiming to differentiate between older cancer patients who are robust enough for standard cancer treatment and those who are more fragile, requiring a subsequent, more thorough assessment to customize their treatment plan accordingly [25].

In addition to its utility as a quick screening instrument for geriatric evaluation, the G8 questionnaire has been recognized as an effective predictor of survival. Multiple research findings have indicated that the G8 can identify functional deterioration and forecast survival in older cancer patients [18].

In two major teaching hospitals in the Netherlands, the G8 and the ISAR-HPs screening tools are regularly used for older lung cancer patients. A study aimed to evaluate the effectiveness of these tools in forecasting patient outcomes, selecting candidates for geriatric assessment, and predicting the likelihood of patients completing treatment. Patients who achieved a normal score on both the G8 and the ISAR-HPs were deemed “fit”. Those with an impaired score on the G8, the ISAR-HPs, or both, were categorized as “potentially frail” and were referred for further evaluation through a geriatric assessment [25].

The study enrolled 142 patients with a median age of 77 years, among whom 84 (59%) were diagnosed with non-small-cell lung cancer, 24 (17%) with small-cell lung cancer, and 5 (4%) with mesothelioma. Out of all participants, 77 (54%) had a malignancy treatable with curative intent based on the tumor’s type, stage, location, and size, while the remaining 65 (46%) were considered for palliative treatment only. Out of the 142 patients studied, 34 (24%) displayed normal results on their frailty screenings, leading to their classification as fit. Conversely, 108 patients (76%) showed impaired screening scores on either the G8 (score ≤ 14) or ISAR-HPs (score ≥ 2), or on both tools, and were thus categorized as potentially frail [25].

One year into the study, 65 out of the 142 patients (46%) had passed away. Of the 34 patients classified as fit, 7 (21%) died, whereas 58 out of the 108 deemed potentially frail (54%) died, demonstrating a statistically significant difference in survival rates between fit and potentially frail patients [25].

Frailty assessment is a crucial component in the treatment of lung cancer, particularly in older patients. Evaluations in the senior population customarily employ screening tools in decision-making, due to the incidence of multiple comorbidities in this population. A comprehensive frailty assessment, including physiotherapy tests and risk stratification questionnaires, is feasible and can aid in treatment planning [93]. Some studies show promise in diagnostic performance evaluation from frailty screening tools, notably the Geriatric 8 (G8) questionnaire and the Groningen Frailty Indicator (GFI) [94,95]. Some authors Loh (2017) emphasized the importance of assessing frailty and vulnerability in older adults with cancer, also suggesting the use of the G8 questionnaire and the GFI [34].

It seems that the GFI is able to differentiate between patients with normal and abnormal comprehensive geriatric assessment (CGA), with a sensitivity of 66% and a specificity of 87% [94]. However, another study reported in 2016 that the GFI had a sensitivity of 76% and a specificity of 73%, which did not meet the predefined minimum of 85% for both sensitivity and specificity. This suggests that the GFI may not be a reliable screening tool for frailty in older patients with lung cancer [95].

Furthermore, there are proposed individualized approaches using Goal Attainment Scaling (GAS) as a reliable, valid, and responsive outcome measure [89].

A series of studies have highlighted the importance of comprehensive geriatric assessment (CGA) in the treatment of senior lung cancer patients. The CGA variables are associated with both function and survival, suggesting that the assessment provides valuable information beyond oncological evaluation [96]. The CGA has a double role in predicting postoperative complications and mortality [97,98]. Furthermore, the CGA can uncover previously unknown health impairments and guide tailored treatment decisions [99].

#### 5.3.2. Other Screening Tools and Assessments

FACT-L and its Lung Cancer Subscale (LCS) have been shown to be effective in assessing quality of life and symptom improvement in lung cancer patients [100]. This questionnaire has been used in various clinical trials, including those for conventional and targeted non-small-cell lung cancer therapy, and has been found to be predictive of tumor response and patient survival [101].

The 6MWT has been found to be valid and reliable in assessing physical function in cancer patients, including those with lung cancer [102]. It has also been used to evaluate the effects of rehabilitation on physical efficiency in lung cancer patients undergoing radiotherapy, with positive changes observed [103]. In the context of lung cancer surgery, the 6MWT has been shown to be useful in assessing the risk of cardiopulmonary complications, with a longer walking distance associated with a lower risk [104]. However, it is important to note that the 6MWT’s performance can be influenced by age, sex, height, and weight, as seen in healthy senior subjects [105].

The Patient-Reported Outcomes Measurement Information System (PROMIS) has been identified as valuable in assessing health-related quality of life in geriatric lung cancer patients [37,106]. This is particularly important in the context of comprehensive geriatric assessment, which has been shown to provide valuable information for the classification and treatment of these patients [107]. The use of patient-reported outcomes (PROs) in lung cancer, including geriatric patients, has been highlighted as essential for patient-centered care and research into comparative effectiveness [108]. However, further research is needed to address measurement gaps, particularly in cases with targeted therapies for advanced-stage lung cancer [109].

The Triage Risk Screening Tool (TRST) is widely recognized for its ease of use and has been validated in numerous settings, demonstrating its effectiveness in improving patient care and optimizing healthcare resources. Its application ranges across different healthcare environments, from emergency departments to inpatient units, aiding in the timely identification of vulnerable patients, but it should not be used as the sole predictor for senior patients’ future healthcare needs [110].

These tools, along with other questionnaires and screening scales, can help physicians assess physical, functional, social, and psychological problems in frail senior patients [111].

## 6. Conclusions

Lung cancer remains one of the most prevalent and lethal cancers worldwide, with a significantly higher incidence and mortality rate among the senior population. 

Senior patients are more susceptible to developing lung cancer due to cumulative exposure to carcinogens and age-related physiological changes. Regular screening and monitoring for lung cancer are crucial in the senior population, facilitating early detection and intervention.

Managing lung cancer in senior patients presents unique challenges, including comorbidities, reduced tolerance to treatment, and varying levels of disease aggressiveness. Treatment plans must be carefully tailored to consider the patient’s overall health, comorbid conditions, and potential side effects in order to ensure the best possible outcomes.

There is a critical need to identify reliable prognostic and predictive biomarkers specifically tailored to the geriatric demographic, in order to optimize treatment strategies and improve outcomes. Incorporating biomarkers like mRNA and epigenetic markers into clinical practice can aid in early detection, prognosis, and monitoring, allowing for more personalized and effective treatment plans.

Geriatric assessment is an important tool for risk stratification and treatment individualization in seniors with lung cancer. Evaluating neuro-cognitive, psycho-emotional, and nutritional status, as well as comorbidities and functional independence, is essential to establishing optimal investigation, treatment, and prognosis plans for senior patients.

In managing lung cancer in senior patients, a holistic approach that goes beyond specific oncological goals is essential. A personalized approach that considers both the patient’s clinical status and their individual needs is essential in the management of senior patients with lung cancer. Baseline assessments and ongoing monitoring are critical in the management of lung cancer in senior patients. Customized treatment strategies incorporating a comprehensive assessment of the patient’s overall health, preferences, and specific cancer characteristics can improve quality of life and their evolution in the face of treatment efficacy.

## Figures and Tables

**Table 1:** Biomarkers used in the diagnosis, prognosis, and treatment of lung cancer.

Acronym	Biomarker	Description
EGFR	Epidermal Growth Factor Receptor	Mutations in the EGFR gene are common in non-small-cell lung cancer (NSCLC) and are important for selecting patients for EGFR tyrosine kinase inhibitor therapy.
ALK	Anaplastic Lymphoma Kinase	Rearrangements in the ALK gene are found in a subset of NSCLC patients, and these patients may benefit from ALK inhibitors.
KRAS	Kirsten Rat Sarcoma Viral Oncogene	KRAS mutations are common in lung cancer and have been associated with resistance to certain therapies, although new treatments targeting specific KRAS mutations are emerging.
ROS1	C-ros Oncogene 1	Like ALK, ROS1 rearrangements can predict responsiveness to specific targeted therapies in NSCLC.
BRAF	Proto-Oncogene B-Raf	Mutations in the BRAF gene, particularly V600E, can be targeted with specific inhibitors in NSCLC.
PD-L1	Programmed Death-Ligand 1	The expression level of PD-L1 can predict the response to PD-1/PD-L1 checkpoint inhibitors in lung cancer.
HER2	Human Epidermal Growth Factor Receptor 2	While less common, HER2 mutations can be targeted with specific therapies in NSCLC.
MET	Mesenchymal–Epithelial Transition	MET exon 14 skipping mutations and MET amplification can influence treatment options in NSCLC.
RET	Rearranged during transfection	Rearrangements in the RET gene can be targeted with RET inhibitors in NSCLC.
P53	Tumor Suppression Gene P 53	Mutations in P53 are common in lung cancer and can have prognostic significance.
TMB	Tumor Mutational Burden	High TMB can predict responsiveness to immunotherapy in some lung cancer settings.
MSI	Microsatellite Instability	Although rarer in lung cancer, these biomarkers can predict response to immunotherapy [11].
dMMR	Mismatch Repair Deficiency	Although rarer in lung cancer, these biomarkers can predict response to immunotherapy [11].

**Table 2:** Geriatric 8 screening tool (G8).

Items	Possible Responses (Score)
Has food intake declined over the past 3 months due to loss of appetite, digestive problems, chewing, or swallowing difficulties?	0 = severe decrease in food intake
	1 = moderate decrease in food intake
	2 = no decrease in food intake
2. Weight loss during the last 3 months?	0 = weight loss > 3 kg
	1 = does not know
	2 = weight loss between 1 and 3 kg
	3 = no weight loss
3. Mobility?	3 = no weight loss
	0 = bed or chair bound
	1 = able to get out of bed/chair but does not go out
	2 = goes out
4. Neuropsychological problems?	0 = severe dementia or depression
	1 = mild dementia
	2 = no psychological problems
5. BMI? (weight in kg)/(height in m^2^)	0 = BMI < 19
	1 = BMI 19 to <21
	2 = BMI 21 to <23
	3 = BMI ≥ 23
6. Takes more than three prescription drugs per day?	0 = yes
	1 = no
7. In comparison with other people of the same age, how does the patient consider his/her health status?	0.0 = not as good
	0.5 = does not know
	1.0 = as good
	2.0 = better
Age	0: >85
	1: 80–95
	2: <80
Total score	0–17

BMI—body mass index.

## Data Availability

No new data were created or analyzed in this study. Data sharing is not applicable to this article.
